# Quality of sickness certification in primary health care: a retrospective database study

**DOI:** 10.1186/1471-2296-14-48

**Published:** 2013-04-12

**Authors:** Ylva Skånér, Britt Arrelöv, Lars G Backlund, Magdalena Fresk, Amanda Waleh Åström, Gunnar H Nilsson

**Affiliations:** 1 Department of Neurobiology, Care Sciences and Society, Centre for Family Medicine, Karolinska Institutet, Stockholm, Sweden; 2 Stockholm County Council, Stockholm, Sweden

**Keywords:** General practitioners, Sick leave, Sickness certificates, Quality indicators, Health care

## Abstract

**Background:**

In the period 2004–2009, national and regional initiatives were developed in Sweden to improve the quality of sickness certificates. Parameters for assessing the quality of sickness certificates in primary health care have been proposed. The aim of this study was to measure the quality of sickness certification in primary health care by means of assessing sickness certificates issued between 2004 and 2009 in Stockholm.

**Methods:**

This was a retrospective study using data retrieved from sickness certificates contained in the electronic patient records of 21 primary health care centres in Stockholm County covering six consecutive years. A total number of 236 441 certificates were used in the current study. Seven quality parameters were chosen as outcome measures. Descriptive statistics and regression models with time, sex and age group as explanatory variables were used.

**Results:**

During the study period, the quality of the sickness certification practice improved as the number of days on first certification decreased and the proportion of duly completely and acceptable certificates increased. Assessment of need for vocational rehabilitation and giving a prognosis for return to work were not significantly improved during the same period. Time was the most influential variable.

**Conclusions:**

The quality of sickness certification practice improved for most of the parameters, although additional efforts to improve the quality of sickness certificates are needed. Measures, such as reminders, compulsory certificate fields and structured guidance, could be useful tools to achieve this objective.

## Background

Sickness certificates issued by physicians are mandatory after one week of self-certification, when a person applies for benefits due to sickness in Sweden. All physicians in Sweden are obliged to issue these certificates. Most of them are issued in general practice [1].

Between 2004 and 2009, there were national and regional initiatives in place to help health care providers handle their responsibility for issuing sickness certificates (Table 1). Furthermore, there has been much focus on the quality of the certificates issued by physicians. The Swedish government has carried out a set of legislative changes in the social security system, such as the introduction of time limited certificates in July 2008. National clinical guidelines with recommended times for sickness certification for different diagnoses were introduced in 2007 to facilitate and standardise physicians’ assessments [2]. Together with educational initiatives tailored for physicians, the measures are expected to reduce the number of individuals on sick leave.

**Table 1:** Activities regarding sickness certification

**Year**	**Regional**	**National**
2004	Regional Social Insurance Medicine Committee	One day education in Social Insurance Medicine for physicians held by Social Insurance Offices
2005	Regional co-operation group; Stockholm County Council and regional Social Insurance Office	
2006	Sick-listing audit^1)^	Economic agreement between the government and the Federation of County Councils during the period 2007-2009^2)^
2007	Regional sick-listing agreement between Stockholm County Council and regional Social Insurance Office^3)^	National sickness certification guidelines
		New form for sickness certification
	Quality parameter project^4)^	
	Educational activities regarding national guidelines	
	Regional sick-listing recommendations^5)^	
2008	Sickness certification audit^1)^	Changes in social insurance regulations
	Education addressed to intern and resident physicians	
2009	Various educational activities	Information from Social Insurance Office regarding changes in Social Insurance regulations

Activities regarding sickness certification in the Stockholm County Council (regional) and national activities and changes in the social insurance legislation and administration (national).

^1^ Medical audit with registration of patient cases by physicians and development of action plans to improve the quality of sickness certification practices.

^2^ Demands regarding activities to increase the county’s accountability with a pecuniary reward dependent on the outcome of regional sickness certification.

^3^ Measures to be taken to meet demands in the national agreement.

^4^Co-operation with other Swedish counties to establish quality parameters regarding completion of sickness certification forms.

^5^The Regional Social Insurance Medicine Committee’s rules of conduct regarding sickness certification.

A report by the Swedish Social Insurance Administration found there was an urgent need to focus on the content of sickness certificates, as they often contain insufficient information [3-6]. National initiatives have been directed towards general practice where the majority of sickness certificates are issued. Differences in the quality of sickness certificates due to context and patient mix have previously been identified [7,8].

The management of a clinic or a primary health care centre (PHCC) is responsible for the quality of the sickness certificates issued by the physicians they employ. To ensure a high quality of sickness certificates, regular monitoring of the sickness certification practices of physicians are necessary.

Since 2004, a group of 27 PHCCs in Stockholm County has been working on bench marking, contributing data and getting feedback regarding various clinical diagnoses and treatments, including sickness certification (the “EK-group”, Efterutbildning och Kvalitet, standing for education and quality). A few of the PHCCs in the network, constituting a “quality group”, have worked on a research project that focuses on the quality of sickness certificates. Between 2008 and 2009, this quality group participated in a joint action with PHCCs from four other Swedish counties to investigate certain quality parameters such as adherence to recommendations on sickness certification from the Regional Social Insurance Medicine Committee of the Stockholm County Council; the sickness certificate forms themselves; knowledge about quality in sickness certification; and the possibility of retrieving data from sickness certificates contained in electronic patient records (EPR). At the beginning the quality control group suggested 18 parameters, which were subsequently tested in a pilot study [9]. Of those 18 parameters, seven were thought to be suitable for highlighting quality aspects in a relevant and measurable way, and were therefore chosen for additional tests. The main selection criteria were face validity, simplicity, and the possibility to determine target levels. Preliminary target levels were also determined for these parameters (except for the parameter “number of days on the first sickness certificate”).

### Aim

The aim of the study was to assess whether the numerous initiatives taken between 2004 and 2009 have resulted in an improvement of sickness certification in primary health care in Stockholm.

## Methods

### Design and setting

This is a retrospective study using data retrieved from the sickness certificates contained in the EPR from 21 PHCCs in Stockholm County covering a 6-year period (2004–2009).

### Population

All PHCCs in Stockholm County work under the same authority, and the 21 participating PHCCs represented different geographical areas, socio-economic characteristics, and numbers of patients and physicians. A total of 236 441 sickness certificates were examined in this study.

### Procedure

In 2010, approximately 200 PHCCs were found in Stockholm County. Of those, 21 were included in the current study. The inclusion criteria were: 1) the possibility to extract EPR data with extraction software MedRave™ (93 PHCCs, all of which were invited to participate), 2) interest in participating, and written consent from the directors of the PHCCs (26 PHCCs), and 3) access to EPR data of at least three of the six years in focus (21 PHCCs). For three of the 21 centres, no data could be retrieved for the period between 2004 and 2006, and for two of the 21 centres, no data could be retrieved for 2007. This was mainly due to privatisation, which made old data inaccessible. The PHCCs did not have to give reasons for not participating in the study. Twelve of the participating 21 PHCCs belonged to the quality network. Extraction software MedRave ™ was used to extract data from EPRs.

### Main outcome measures

Table 2 shows the seven quality parameters used as outcome variables in this study. The preliminary target levels are only briefly referred to in this report.

**Table 2:** Definition of the quality parameters

**Quality parameter**	**Explanation**
1. Number of days on the first sickness certificate during a sickness absence episode issued within the centre.^1)^	This has been shown to be an important predictor of total length of the sickness absence period.
2. Proportion of sickness certificates issued on the basis of a face-to-face consultation between issuing physician and patient.^2)^	Sickness certificates should in general be issued on the basis of a face-to-face consultation.
3. Proportion of non-specific diagnoses (ICD-10 R or ICD-10 Z) after 30 days of sickness absence.^3)^	Non-specific diagnoses may be adequate during the first part of a sickness absence episode, but after 30 days the patient should have a specific ICD-10 diagnosis.
4. Proportion of sickness certificates with documented assessment of need/no need for vocational rehabilitation after 30 days of sick leave.^3)^	After 30 days of sickness absence, the physician should make an evaluation of the patient’s need for vocational rehabilitation.
5. Proportion of sickness certificates with documentation about prognosis regarding scheduled time for return to work after 30 days of sickness absence.^3)^	After 30 days of sickness absence, there should be an evaluation of the patient’s prognosis regarding return to work.
6. Proportion of completely filled in sickness certificates (13 specified fields of information filled in) after 30 days of sickness absence.^3)^	This is a criterion required by the Social Insurance Offices.
7. Proportion of sickness certificates with the minimum amount of information filled in: medical history, examination and functional limitations.^2)^	This is a criterion considered by the quality group as a minimum requirement for all sickness certificates.

^1^Based on the first sickness certificates issued in the PHCC during the actual sickness absence episode.

^2^Based on all sickness certificates;

^3^Based on the first sickness certificates issued after the first 30 days of a sickness absence episode.

### Analysis

Since ensuring sickness certification of high quality is the responsibility of the director of a PHCC, the centres themselves were used to measure changes over time for each of the seven quality parameters. Medians for parameter 1 and proportions for parameters 2–7 were calculated for each PHCC. The overall medians for each parameter were calculated for all PHCCs. The medians were chosen instead of the arithmetic means because the number of patients varied widely across the different PHCCs and because medians are less influenced by outliers than are arithmetic means. First and third quartiles were also calculated.

For the regression models, all sickness certificates were used but PHCCs were not included as outcome variables. Explanatory factors of time, sex and age group were used to generate the complete regression model. In Step 1, complete regression models were generated with the seven parameters as outcome variables and time (years 2004–2009), sex and age group (20–29, 30–39, 40–49, 50–59 and 60+) as explanatory variables. In Step 2, the variables that were significant in the complete models were used to generate the final models. The coefficient of determination (R^2^) is the proportion of variance explained by a model.

The number of certificates was large enough to generate a model significant enough for each parameter. This way, it was possible to understand how each explanatory factor affected the dependant variable.

The statistical software used was SAS 9.2/Enterprise Guide.

## Results

### Changes in quality over time

**Figure 1:**
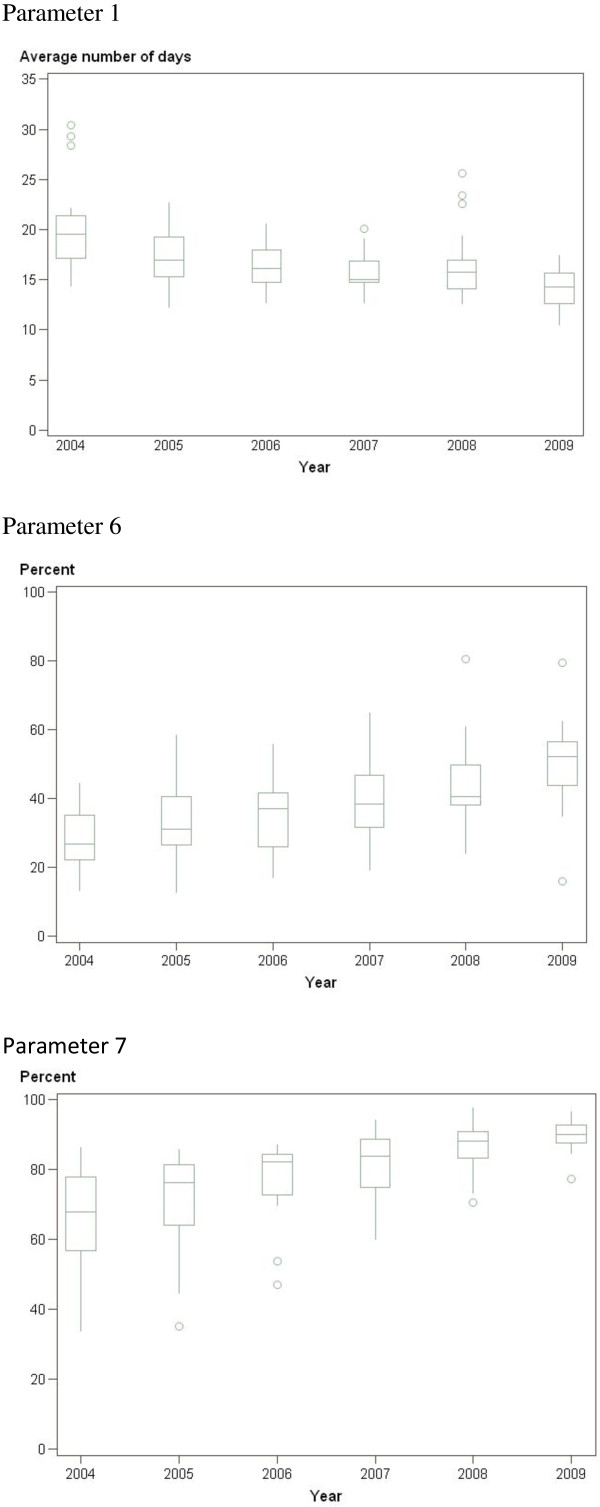
**Box plots for parameters 1, 6 and 7.** Box plots for Number of days on first sickness certificate (parameter 1), Proportion of completely filled in certificates after 30 days (parameter 6) and Proportion of acceptable certificates (parameter 7). The outcome variables are presented as box plots with medians, minimum and maximum values, and lower and upper quartiles (25^th^ percentile, Q1, and 75^th^ percentile, Q3) for outcome variables in models with R^2^ > 20%. On the Y-axis: Average number of days for parameter 1, percent for parameters 6 and 7. On the X-axis: Years for all three parameters.

The median number of days of sick leave on the first sickness certificate issued by a PHCC (parameter 1) was reduced from 19 to 14 during the study period (Table 3 and Figure 1). The proportion of certificates issued after face-to-face contact (parameter 2) increased from 79% to 90% (Table 2). The proportion of certificates with non-specific diagnoses (parameter 3), which should have been low, ranged between 8% and 10% and did not vary significantly during the study period (Table 3). The proportion of certificates with documented assessment of need for vocational rehabilitation (parameter 4) increased from 44% to 59%, and the proportion with documented prognosis for return to work (parameter 5) increased from 70% to 85% during the same period (Table 3). The proportion of duly completed certificates (parameter 6) increased from 27% to 52% (Table 3 and Figure 1) and the proportion of acceptable certificates (parameter 7) increased from 68% to 90% (Table 3 and Figure 1). The differences between the members and the non-members of the quality network were a few percent units for parameters 1–3 and 6–7 for all the years. Non-members’ results were about ten percent units better for all the years for parameter 5 and for the last two years for parameter 4.

**Table 3:** Medians of quality parameters per year for all PHCCs

**Quality parameter**	**2004 n = 18**	**2005 n = 18**	**2006 n = 18**	**2007 n = 19**	**2008 n = 21**	**2009 n = 21**
1. Number of days on first certificate (days)	19	17	16	15	15	14
	(17; 20)	(15; 19)	(15; 17)	(14; 17)	(14; 17)	(13; 15)
2. Proportion of face-to-face consultations (%)	79	81	81	83	87	90
	(74; 86)	(76; 86)	(76; 85)	(77; 86)	(82 90)	(86; 92)
3. Proportion of certificates with non-specific diagnoses after 30 days (%)	10	10	8	9	10	8
	(8; 13)	(7; 11)	(7; 13)	(8; 11)	(8; 12)	(5; 10)
4. Proportion of certificates with notation about need for vocational rehabilitation after 30 days (%)	44	51	45	49	58	59
	(37; 46)	(44; 56)	(37; 58)	(46; 62)	(49; 64)	(52; 73)
5. Proportion of certificates with notation about prognosis for return to work after 30 days (%)	70	65	68	72	79	85
	(61; 76)	(55; 71)	(62; 75)	(63; 80)	(71; 83)	(79; 93)
6. Proportion of completely filled in certificates after 30 days (%)	27	32	36	38	41	52
	(21; 35)	(25; 40)	(23; 40)	(31; 47)	(38; 50)	(44; 60)
7. Proportion of acceptable certificates (%)	68	76	82	84	88	90
	(56; 77)	(63; 82)	(71; 84)	(74; 89)	(84; 90)	(87; 92)

Medians of medians and quartile values (Q1; Q3) are shown**;** n = number of PHCCs.

### Regression models for the outcome variables

The final regression models with the quality parameters as outcome variables are shown in Table 4. Time was a significant variable in all models except for those based on certificates with non-specific diagnoses (parameter 3). Time was also the only significant variable in the models based on certificates with documented prognosis for return to work (parameter 5) and acceptable certificates (parameter 7), and, together with age group, it was included in the models for all other parameters. Sex was a significant variable only in the model for parameter 3 (non-specific diagnoses; more common in women) but this model is less suitable. Age group was the most significant variable in the model for parameter 1 (number of sick leave days on the first sickness certificate). That being said, advancing age was shown to influence negatively the quality of sickness certification.

**Table 4:** Variation in quality explained by regression models

**Quality parameter**	**Model description**	**Parameter estimates**					
**Y variable**	**R**^**2**^	**X variable**	**Regression coefficient**	**t-value**	**Pr > |t|**	**95% Confidence limits**	
					**Sign parameter**	**Lower limits**	**Upper limits**
1. Number of days on first certificate	29.5%	Time	−0.94	−10.96	<.0001	−1.10	−0.77
		Age group	1.97	18.96	<.0001	1.77	2.17
2. Face-to-face meeting	15.6%	Time	0.02	13.24	<.0001	0.02	0.02
		Age group	−0.01	−6.06	<.0001	−0.01	−0.01
3. Non-specific diagnoses	1.14%	Sex	0.02	3.62	0.0003	0.01	0.04
4. Documentation of need for vocational rehabilitation	5.7%	Time	0.03	7.84	<.0001	0.02	0.04
		Age group	−0.01	−2.80	0.0052	−0.02	−0.00
5. Documentation of prognosis	7%	Time	0.03	9.25	<.0001	0.03	0.04
6. Complete certificates	27.6%	Time	0.04	20.48	<.0001	0.04	0.05
		Age group	−0.01	−4.13	<.0001	−0.02	−0.01
7. Acceptable certificates	34%	Time	0.05	24.53	<.0001	0.04	0.05

Variation in quality explained by models with the seven parameters as dependant variables (y) and time, sex and age group as explanatory variables (x).

The models for parameter 1 (number of sick leave days on the first sickness certificate), parameter 6 (duly completed certificates) and parameter 7 (acceptable certificates) were the strongest models, with an R^2^ of approximately 30%, while the model for parameter 2 (face-to-face contacts) had an R^2^ of 15.6%, which indicated relative strength. By contrast, models based on certificates with non-specific diagnoses (parameter 3), documented assessment of need for vocational rehabilitation (parameter 4), and documented prognosis for return to work (parameter 5) were found to be very weak, at least partly because of skewed distribution of the data.

## Discussion

In the current study we observed an increase in the quality of sickness certification between 2004 and 2009 in primary health care in Stockholm County by means of examining all sickness certificates issued during that period. Time was the decisive factor, and accounted for the variability observed in three of the seven parameters assessed, i.e. number of days on the first certificate, completely filled in certificates and acceptable certificates, and to some extent for one of the parameters, namely face-to-face consultations.

There are few studies that have examined the parameters in the current study, and no other study has measured changes in sickness certification over time. Information about the need for vocational rehabilitation after 30 days of absence was lacking in 60% of the certificates in a Swedish study of general practitioners’ certificates from 2002 [3]. With regard to information about prognosis for return to work after 30 days of absence, two Swedish studies [3,10] showed that it was lacking in 29% and 27% of the certificates, respectively. These results were consistent with our findings from 2004. Notes on medical history, physical examination and functional limitations in the sickness certificates comprised a compound measure in the current study and may therefore be difficult to compare to findings from previous studies. Nevertheless, in an earlier Swedish study, information about physical examination was missing in 35% of certificates, and 52% of these contained unclear information about functional capacity, which is similar to our results [3].

The reduction in the number of days on the first sickness certificate that was observed in this study over time was paralleled by a decrease in the total number of sickness days in the population during the same period. The median number of days of fulltime sickness absence per 10 000 inhabitants attending the 21 PHCC was 31 830 in 2004 and 19 769 in 2009 (data presented in a report in Swedish) [11]. The rise in the proportion of face-to-face consultations over time may have been influenced by financial changes that affected PHCCs in 2007 when funds to conduct telephone consultations were withdrawn.

The main strength of this study was its use of authentic data retrieved directly from the medical records in 21 PHCCs of Stockholm County covering six consecutive years, and the large sample population, consisting of more than 200 000 sickness certificates. Although a number of factors were shown to affect sickness certification, we cannot exclude the possibility that other explanatory factors may have been involved which were not studied here.

Since 2008, time limits have been used in the Swedish social security system. This has made parameters 4–7 more important than ever before. Information about the need for vocational rehabilitation and prognosis for return to work (parameters 4 and 5) is necessary for the implementation of measures taken by the employer and the social insurance officer to support persons on sick leave who wish to return to work. Complete and detailed information (parameter 6) is required by the Social Insurance Offices after 30 days of sickness absence, while a minimum amount of information (parameter 7) is needed in all cases. Overall, better planning is required to avoid long, drawn-out benefit payments. We found a considerable discrepancy between preliminary target quality levels and actual quality levels in relation to information about the need for vocational rehabilitation and complete certificates. This discrepancy implies that additional efforts would be needed to improve the quality of those two parameters.

The fact that the PHCCs in the quality network did not get better results than the others may indicate that getting feedback on sickness certification is not enough to improve sickness certification; local quality work is needed as well. Overall, GPs in many countries regard sickness certification as a complex task [12-14].

Educational initiatives have been, by far, the most commonly used way to bring about change in physicians’ sickness certification practices, but their effect on the quality of certificates issued was found to have been rather small, perhaps due to insufficient or inadequate focus [15]. Complementary measures would be required to change physicians’ sickness certification practices. One such measure could be the use of reminders, which has been shown to be successful, for example, in safe drug prescriptions [16]. Another tool might be electronic completion and on-line transmission of the sickness certificate, which is introduced in other parts of Sweden as well as in Italy [17]. Using this method, filling in certain fields could be made compulsory, which would automatically affect parameters 4–7 in this study.

A parameter not addressed in this study but which may have affected quality of sickness certificates is problems physicians may have in making the necessary assessments. This has been demonstrated in earlier Swedish studies as well in studies from other countries [12,14,18]. To resolve this, the WHO International Classification of Functioning Disability and Health (ICF) has been discussed as a possible tool. In Sweden, measures have been taken to use ICF codes in a structured way that is integrated into the electronic sickness certificate system. A pilot study of ours conducted in Stockholm County implied that the use of ICF, via ICF Core Sets, could be a way to increase the quality of the descriptions of activity limitations, an area where improvements are urgently needed (submitted).

## Conclusions

The quality of the sickness certification practice improved over time during the study period with respect to the number of days on first certification and the proportion of properly completed and acceptable certificates but assessment of the need for vocational rehabilitation and giving a prognosis for return to work were not significantly improved over time during the same period. Although the quality levels of all these parameters were not satisfactory they may provide a good basis for further work. To improve the quality of sickness certificates, measures, such as reminders, compulsory certificate fields, and structured guidance, would be needed.

## Competing interests

The authors declare that they have no competing interests.

## Authors’ contributions

YS, BA, LB and GHN participated in the study design. AWÅ performed the data management and statistical analyses. YS drafted the manuscript. All authors participated in the interpretation of data and reviewed the manuscript. GHN is the guarantor. All authors read and approved the final manuscript.

## Authors’ information

YS has many years of experience as a specialist in General Practice, and has a PhD including studies on Medical Judgement and Decision-making. She holds a position as Associated Professor at Karolinska Institutet, Stockholm, Sweden.

BA has many years of experience as a specialist in General Practice, and has a PhD that included studies on sickness certification. She holds a position as Senior Consultant and Medical expert in Social Insurance Medicine, Stockholm County Council, Sweden.

LGB has many years of experience as a specialist in General Practice. He has a PhD that included studies on Medical Judgement and Decision-making and holds a position as a researcher at Karolinska Institutet, Stockholm, Sweden.

MF is a specialist in General Practice. She has developed a web–based tool for using ICF as a tool in the sickness certification process.

AWÅ is a statistician and has been working for many years with large data bases in Stockholm County Council, Sweden.

GHN is a specialist in General Practice, and has a PhD that included studies in informatics and decision-making. He holds a position as professor at Karolinska Institutet, Stockholm, Sweden.

All authors are members of a research group at the Centre for Family Medicine, Karolinska Institutet that works with and plans a number of studies related to the quality of sickness certification.

## Pre-publication history

The pre-publication history for this paper can be accessed here:

http://www.biomedcentral.com/1471-2296/14/48/prepub
